# Insight into S-RNase-based self-incompatibility in *Petunia*: recent findings and future directions

**DOI:** 10.3389/fpls.2015.00041

**Published:** 2015-02-05

**Authors:** Justin S. Williams, Lihua Wu, Shu Li, Penglin Sun, Teh-Hui Kao

**Affiliations:** ^1^Department of Biochemistry and Molecular Biology, Pennsylvania State University, University Park, PA, USA; ^2^Intercollege Graduate Degree Program in Plant Biology, Pennsylvania State University, University Park, PA, USA

**Keywords:** *Petunia*, SCF^SLF^ complex, self-incompatibility, S-locus F-box protein, Solanaceae, S-RNase

## Abstract

S-RNase-based self-incompatibility in *Petunia* is a self/non-self recognition system that allows the pistil to reject self-pollen to prevent inbreeding and to accept non-self pollen for outcrossing. Cloning of *S-RNase* in 1986 marked the beginning of nearly three decades of intensive research into the mechanism of this complex system. S-RNase was shown to be the sole female determinant in 1994, and the first male determinant, S-locus F-box protein1 (SLF1), was identified in 2004. It was discovered in 2010 that additional SLF proteins are involved in pollen specificity, and recently two *S*-haplotypes of *Petunia inflata* were found to possess 17 *SLF* genes based on pollen transcriptome analysis, further increasing the complexity of the system. Here, we first summarize the current understanding of how the interplay between SLF proteins and S-RNase in the pollen tube allows cross-compatible pollination, but results in self-incompatible pollination. We then discuss some of the aspects that are not yet elucidated, including uptake of S-RNase into the pollen tube, nature, and assembly of SLF-containing complexes, the biochemical basis for differential interactions between SLF proteins and S-RNase, and fate of non-self S-RNases in the pollen tube.

## INTRODUCTION

Self-incompatibility (SI) is a pre-zygotic reproductive barrier, which prevents inbreeding in many families of angiosperms (Takayama and Isogai, 2005; Franklin-Tong, 2008). *Petunia* possesses the Solanaceae type SI in which this reproductive barrier is regulated by the highly polymorphic *S*-locus. The *S*-locus houses the female determinant gene, *S-RNase* (Lee et al., 1994; Murfett et al., 1994), and multiple male determinant genes, *S-locus F-box* (*SLF*) genes (Sijacic et al., 2004; Kubo et al., 2010). In *Petunia*, 32 *S*-haplotypes have been reported (Sims and Robbins, 2009). A diploid pistil carries two different *S*-haplotypes, each producing an allelic variant of S-RNase. S-RNase is synthesized in the transmitting tissue of the style and secreted into the transmitting tract where pollen tubes grow from the stigma to the ovary. A pollen tube takes up both self S-RNase (product of the same *S*-haplotype as that carried by pollen) and non-self S-RNase (product of a different *S*-haplotype from that carried by pollen; Luu et al., 2000; Goldraij et al., 2006); however, only self S-RNase can inhibit the growth of the pollen tube (in the upper one-third of the style) through its RNase activity (Huang et al., 1994).

The understanding of how a pollen tube escapes the cytotoxic effect of non-self S-RNase has undergone several major developments and revisions in the past decade. SLF was first discovered in *Antirrhinum hispanicum* (Lai et al., 2002; Qiao et al., 2004b), which possesses the same type of SI. Subsequently, the first SLF in *Petunia*, now named SLF1, was confirmed as a male determinant via an *in vivo* functional assay (Sijacic et al., 2004). The presence of an F-box domain in the N-terminal region of SLF led to the proposal that SLF, like conventional F-box proteins, is a component of a class of E3 ubiquitin ligase, the SCF (Skp1-Cullin1-F-box protein) complex, involved in ubiquitin-mediated protein degradation by the 26S proteasome (Lai et al., 2002; Qiao et al., 2004a; Hua and Kao, 2006). The substrate of an SCF^SLF^ complex appears to be non-self S-RNase(s) for the specific allelic variant of SLF in the complex, as an *in vitro* protein pull-down assay showed that non-self interactions between allelic variants of SLF and S-RNase were stronger than self-interactions. This could explain why only self S-RNase can exert a cytotoxic effect on the pollen tube, as it is not ubiquitinated or degraded in the pollen tube. However, given that there are a large number of *S*-haplotypes in *Petunia* and given that allelic variants of S-RNase exhibit a high degree of sequence diversity, it is difficult to envision how an allelic variant of SLF could interact with so many non-self S-RNases, but not with a single self S-RNase. This conundrum was solved when it was discovered that at least two paralogous genes of *SLF1* are also involved in pollen specificity (Kubo et al., 2010). A new model, “collaborative non-self recognition,” proposes that multiple SLF proteins produced by pollen of a given *S*-haplotype collaboratively recognize and detoxify all non-self S-RNases (i.e., each SLF is only capable of interacting with a subset of its non-self S-RNases), but none can interact with their self S-RNase (Kubo et al., 2010). To date, *S_2_*-haplotype and *S_3_*-haplotype of *Petunia inflata* have been shown to possess the same 17 *SLF* genes based on pollen transcriptome analysis (Williams et al., 2014b). Moreover, eight other *Petunia S*-haplotypes have recently been shown to possess 16–20 *SLF* genes (Kubo et al., 2015). So far, eight of them (*SLF1*, *SLF2*, *SLF3*, *SLF4*, *SLF5*, *SLF6*, *SLF8*, and *SLF9*) have been confirmed by an *in vivo* functional assay to be involved in pollen specificity (Sijacic et al., 2004; Kubo et al., 2010, 2015; Williams et al., 2014a).

**FIGURE 1:**
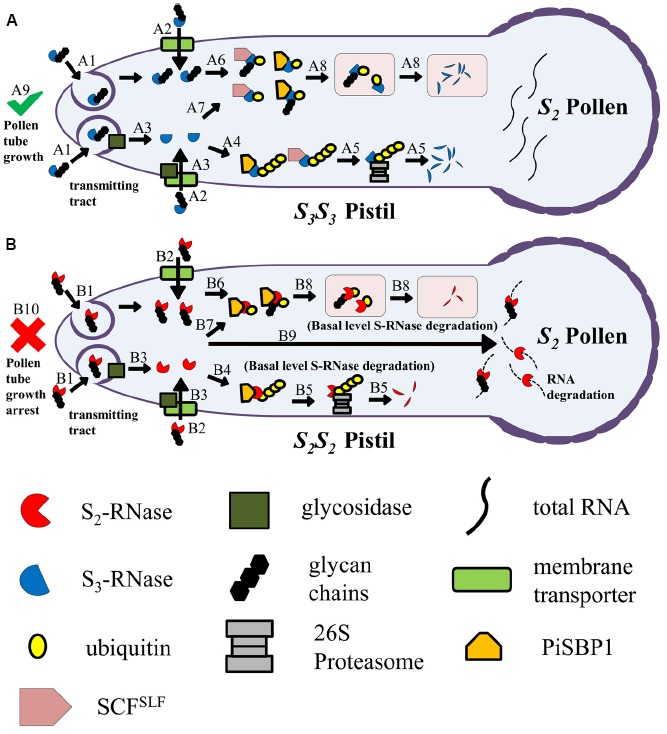
**Model for uptake of S-RNase by the pollen tube in the transmitting tract of the pistil, and fates of self and non-self S-RNases after uptake.** **(A)** An *S_2_* pollen tube, growing in an *S_3_S_3_* pistil, takes up S_3_-RNase (a non-self S-RNase). Two possible types of uptake mechanisms are depicted: clathrin-dependent or clathrin-independent endocytosis (A1) and membrane-transporter mediated (A2). During uptake, the N-linked glycan chains of S_3_-RNase may be removed by a membrane-associated glycosidase (A3). The deglycosylated S_3_-RNase becomes poly-ubiquitinated (A4), mediated largely by the conventional SCF^SLF^ complex and to a much lesser extent by PiSBP1 and the PiSBP1-contaning novel SCF^SLF^ complex (not shown). The poly-ubiquitinated S_3_-RNase is destined for degradation by the 26S proteasome (A5). S_3_-RNase may remain glycosylated and be mono-ubiquitinated (A6), again mediated largely by the conventional SCF^SLF^ complex and to some extent by PiSBP1 and the PiSBP1-contaning novel SCF^SLF^ complex (not shown). The deglycosylated S_3_-RNase may also be similarly mono-ubiquitinated (A7). The mono-ubiquitinated (deglycosylated) S_3_-RNase is then targeted to vacuoles or vacuole-like organelles for degradation (A8). All the steps depicted result in detoxification of the S_3_-RNase molecules inside the *S_2_* pollen tube, allowing it to reach the ovary to effect fertilization (A9). **(B)** An *S_2_* pollen tube, growing in an *S_2_S_2_* pistil, takes up S_2_-RNase (self S-RNase). S_2_-RNase is taken up by the same mechanisms (B1 and B2) as those depicted for S_3_-RNase in **(A)**, and may also be subjected to deglycoslyation (B3). None of the SLF proteins in the SCF^SLF^ complexes are able to interact with their self S-RNase to mediate its degradation or compartmentalization. However, similar to the scenarios depicted in **(A)**, PiSBP1 may mediate poly-ubiquitination of S_2_-RNase (B4) for basal-level degradation by the 26S proteasome (B5), and may mediate mono-ubiquitination of both S_2_-RNase (B6) and deglycosylated S_2_-RNase (B7). The mono-ubiquitinated (deglycosylated) S_2_-RNase is then targeted to vacuoles or vacuole-like organelles for degradation (B8). However, the majority of S_2_-RNase molecules remain intact and will degrade RNA (B9) to result in growth arrest of the pollen tube (B10). Note: To make it easier to follow the different fates of self and non-self S-RNase, *S_2_S_2_* and *S_3_S_3_* pistils are used in this figure; however, in nature, pistils of a self-incompatible species are normally heterozygous for the *S*-locus.

Despite the impressive progress made in understanding the complex non-self recognition between the female determinant and multiple male determinants, there are still many aspects of S-RNase-based SI that remain unknown. In this article, we discuss the current understanding of some of these aspects, summarize the key features of the discussion in the model shown in Figure 1, and list the known and putative proteins involved in Table 1.

**Table 1:** **Known and putative proteins involved in S-RNase-based self-incompatibility**.

**Component of SI process**	**Known proteins**	**Putative proteins**
S-RNase uptake	S-RNase	Membrane transporters, endocytosis-related proteins
SCF^SLF^ complex	PiCUL1-P, PiSSK1, SLF proteins, PiRBX1	PiSBP1
Novel SCF^SLF^ complex		SLF1, PiCUL1-G, PiSBP1
S-locus F-box proteins	SLF1–SLF17	
Fate of non-self S-RNase in the pollen tube	SCF^SLF^ complex, 26S proteasome	Glycosidase, PiSBP1, PiCUL1-G, vacuolar proteases

## UPTAKE OF S-RNase INTO POLLEN TUBES

For S-RNase to exert its cytotoxic effect it must be taken up by the pollen tube in the transmitting tract; however, the mechanism of this critical step in SI is not yet known. S-RNase could be taken up by endocytosis and/or membrane transporters, both of which are used by the pollen tube to take up extracellular proteins (Moscatelli et al., 2007; Chen et al., 2010).

A delicate equilibrium of exocytosis and endocytosis is required for the rapid polarized growth of the pollen tube (Camacho and Malhó, 2003). Both clathrin-dependent and clathrin-independent endocytosis have been shown to be involved in recycling plasma membrane proteins/lipids and in regulating pollen tube growth (Onelli and Moscatelli, 2013). One notable example is the uptake of SCA (stigma/style cysteine-rich adhesin), a protein that guides the pollen tube toward the ovary; it is internalized in the tip region of a growing pollen tube through clathrin-dependent endocytosis and sorted to the multi-vescular bodies and vacuoles (Kim et al., 2006; Chae et al., 2007).

ATP-binding cassette (ABC) transporters constitute a large protein family in plants (Geisler, 2014), and have been reported to be involved in various processes, e.g., pathogen attack response, deposition of plasma membrane lipids, nutrient accumulation in seeds, and transport of phytohormones (Kang et al., 2010; Tunc-Ozdemir et al., 2013). ABC transporters are active transporters, deriving energy from ATP hydrolysis, and they are either exporters or importers (Geisler, 2014). In plants, ABC transporters have been classified into 13 subfamilies (Rea, 2007). An ABC transporter of apple, MdABCF, has been implicated in the transport of S-RNase into the pollen tube (Meng et al., 2014). Analysis of the *S_2_*-pollen, *S_3_*-pollen, and *S_3_S_3_* leaf transcriptomes of *P*. *inflata* (Williams et al., 2014b) reveals the presence of 476, 334, and 851 BLAST annotated transporters, and interestingly, ∼59% of the potential transporters in each pollen transcriptome are annotated as ABC transporters, whereas only ∼25% of the potential transporters in the leaf transcriptome are annotated as ABC transporters (our unpublished data). Given that S-RNase is pistil-specific and is taken up only by the pollen tube, if ABC transporters are involved in the uptake of S-RNases, they would likely be among those that are pollen-specific.

Regardless of how S-RNase is taken up by the pollen tube, the uptake machinery must be able to interact with a large number of highly divergent allelic variants of S-RNase. Despite the sequence diversity, S-RNase contains five conserved regions (C1–C5; Ioerger et al., 1991). Alignment of the amino acid sequences of 20 allelic variants of S-RNase in *Petunia* available in the NCBI non-redundant nucleotide database reveals 21 perfectly conserved amino acid residues. As the crystal structure of *Nicotiana alata* S_F11_-RNase has already been determined (Ida et al., 2001), those conserved residues that are exposed on the outside surface would be good candidates to use for investigating their role in interaction with a transporter and/or a transmembrane receptor involved in uptake.

## THE SCF^SLF^ COMPLEX

The SLF-containing SCF complex, SCF^SLF^, of *P*. *inflata* has been shown by co-immunoprecipitation (Co-IP) followed by mass-spectrometry (MS) to contain a conventional Rbx1 (PiRBX1; a RING-finger protein), a pollen-specific Cullin1 (PiCUL1-P), and a pollen-specific Skp1-like protein (PiSSK1; Li et al., 2014). Similar components have been identified in the SCF complex of *Petunia hybrida* (Entani et al., 2014; Liu et al., 2014) and *Pyrus bretschneideri* (Xu et al., 2013). Phylogenetic studies showed that SSK1 and Cullin1 proteins implicated in SI form their own monoclades (Xu et al., 2013; Yuan et al., 2014), and that the 17 SLF proteins of *P*. *inflata* form a monoclade. Also, SLF proteins were the only F-box proteins that co-immunoprecipitated with PiSSK1 (Li et al., 2014). Interestingly, tomato SpCUL1, sharing 91% sequence identity with PiCUL1-P, is involved in unilateral incompatibility between tomato species (Li and Chetelat, 2010) and is also required for compatible pollination in *Solanum arcanum* (Li and Chetelat, 2013). Thus, three of the four components of the SCF^SLF^ complex (PiSSK1, PiCUL1-P, and SLF) appear to have evolved specifically to function in SI.

A *P*. *hybrida* RING-finger protein, termed PhSBP1, was found to interact with S-RNase by a yeast two-hybrid assay (Sims and Ordanic, 2001). Subsequently, the PhSBP1 homolog from *P*. *inflata*, termed PiSBP1, was found to interact with an SLF protein (S_2_-SLF1), a different Cullin1 (PiCUL1-G) and S-RNase by a yeast two-hybrid assay (Hua and Kao, 2006; Meng et al., 2011), leading to the suggestion that PiSBP1 plays the roles of both Skp1 and Rbx1, and forms a novel SCF complex with PiCUL1-G and SLF1 (Hua and Kao, 2006). In *Malus* × *domestica* (apple), both homologs of PiSBP1 and PiSSK1 were found to interact with an SLF (named SFB for S-Locus F-Box) and a Cullin1 (MdCUL1) by an *in vitro* protein binding assay (Minamikawa et al., 2014). MdSSK1 interacted with the SLF protein more strongly than did MdSBP1, and the transcript level of *MdSSK1* was >100 times higher than that of *MdSBP1*. Thus, the conventional SCF^SLF^ complex is thought to play a major role in mediating ubiquitination and degradation of non-self S-RNases. This finding may explain why PiSBP1 was not identified from the Co-IP products using either S_2_-SLF1 or PiSSK1 as bait (Li et al., 2014). PiSBP1 may also function as a mono-subunit E3 ubiquitin ligase as it catalyzed ubiquitination of S_3_-RNase in the presence of E1, E2, and ubiquitin in an *in vitro* assay (Hua and Kao, 2008). However, the interaction of PiSBP1 with S-RNase does not show allele specificity, so it may mediate basal level S-RNase degradation, perhaps as a safety mechanism to ensure that all non-self S-RNases are cleared from the pollen tube.

The approach of using Co-IP followed by MS has been successful in identifying three (SLF1, SLF4, and SLF13) of the 17 SLF proteins of *P*. *inflata* as the F-box component of the SCF^SLF^ complexes (Li et al., 2014). Among these 17 SLF proteins, eight (including SLF1 and SLF4) have so far been confirmed to be involved in pollen specificity via an *in vivo* functional assay (Sijacic et al., 2004; Williams et al., 2014a; Kubo et al., 2015). This assay involves raising transgenic plants to examine the effect of expressing a particular allelic variant of an SLF on the SI behavior of the transgenic pollen. In cases when breakdown of SI is observed in the transgenic plants, progeny from crosses with wild-type plants of appropriate *S*-genotypes have to be examined as well. The Co-IP-MS results suggest that this approach may be a much less time-consuming and labor-intensive alternative to the *in vivo* functional assay for assessing the SI function of SLF proteins. If all SLF proteins are assembled into similar SCF complexes, this would raise the questions of how all these sequence-divergent SLF proteins (e.g., 45.3–87.7% sequence identity between the 17 SLF proteins of *S_2_*-haplotype; Williams et al., 2014b) are capable of being assembled into their respective SCF^SLF^ complexes, and whether the allelic variants of S-RNase taken into a pollen tube may favor the “selection” of particular SLF proteins that can interact with and detoxify the non-self S-RNases, especially when the common components of the complex are limited. It would be interesting to study as well the dynamics of the SLF-containing SCF complexes during growth of compatible pollen tubes in the pistil.

## THE S-LOCUS F-BOX PROTEINS

For the SLF proteins of *Petunia* that have been studied so far, each interacts with only one, or a few, of the S-RNases examined (Sijacic et al., 2004; Kubo et al., 2010; Williams et al., 2014a). This pattern of interactions between SLF proteins and S-RNases is consistent with the prediction by the collaborative non-self recognition model (Kubo et al., 2010). How then does one SLF, but not other SLF proteins, interact with a certain S-RNase? How have all these multiple SLF proteins evolved so that pollen of a given *S*-haplotype has a complete arsenal to counter the cytotoxic effect of all non-self S-RNases, but avoid interacting with their self S-RNase?

Typically, F-box proteins contain two domains: the F-box domain in the N-terminal region and a protein–protein interaction domain located in the C-terminal region (C-terminal domain or CTD; Gagne et al., 2002; Cardozo and Pagano, 2004; Wang et al., 2004). Thus, it is reasonable to examine the CTD of an SLF to identify the amino acids that are involved in its interaction with a particular S-RNase. One approach to identify such amino acids would be to compare the amino acid sequences of SLF proteins that interact with the same S-RNase with the amino acid sequences of SLF proteins that do not. The amino acids in the CTD that are conserved among all the SLF proteins that interact with the same S-RNase, but are divergent among all those that do not, are likely important for the specific interaction with that S-RNase. This approach will benefit from knowing interaction relationships between as many SLF proteins and S-RNases, as the information can then be used to design strategies to determine the biochemical basis for the differential interactions.

Among the interaction relationships established between SLF proteins and S-RNases of *P*. *inflata*, S_2_-SLF1 interacts with the largest number, four, of the S-RNases examined and all the other SLF proteins interact with none or at most one S-RNase (Sijacic et al., 2004; Kubo et al., 2010; Sun and Kao, 2013; Williams et al., 2014a). The SLF that interacts with more S-RNases than do all other SLF proteins might be the first to have come into existence during the evolution of the SI system. If the first SLF could interact with a number of non-self S-RNases, it would allow pollen to detoxify new non-self S-RNases as more *S*-haplotypes evolved, without having to generate a new SLF. However, there might be a practical limit as to the number of non-self S-RNases with which each SLF could interact, so that when the maximum capacity is reached, a new SLF would be needed to allow pollen to recognize and detoxify additional non-self S-RNases as more *S*-haplotypes continued to evolve.

If an SLF has evolved to interact with and detoxify a particular S-RNase, there would be no selective pressure to generate another SLF with the same function. However, from the standpoint of defense against the toxic effect of non-self S-RNases, it would be beneficial to pollen if more than one SLF were capable of detoxifying any particular non-self S-RNase, as this will minimize the deleterious effect caused by mutations that render an SLF incapable of interacting with and detoxifying a non-self S-RNase. In order to maintain an SI system over a long period of time, not only must self-pollen be rejected by the pistil, but also non-self pollen must be accepted by the pistil through the collective effort of all SLF proteins to detoxify all non-self S-RNases. It would be of interest to determine whether, during the evolution of the *SLF* genes, there has been indeed such a redundancy built in for pollen to deal with every non-self S-RNase. The results from studying the effect of silencing the expression of S_2_-SLF1 in pollen of *S_2_S_3_* transgenic plants are consistent with the presence of additional SLF proteins for detoxifying S_3_-RNase, S_7_-RNase, and S_13_-RNases, as transgenic pollen producing very low levels of, if any, S_2_-SLF1, remained compatible with *S_3_*-, *S_7_*-, and *S_13_*-carrying pistils (Sun and Kao, 2013). Moreover, Kubo et al. (2010) found that two SLF proteins produced by *S_5_* pollen of *P*. *hybrida* interacted with the same S-RNase, S_9_-RNase.

## THE FATE OF NON-SELF S-RNases IN THE POLLEN TUBE

The current view about what happens to non-self S-RNases, after being taken up by the pollen tube, is that they become ubiquitinated in the cytosol through mediation by appropriate SCF^SLF^ complexes, and subsequently degraded by the 26S proteasome. However, the fate of non-self S-RNases may need a closer examination, considering the following observations in *Petunia*. A cell-free protein degradation system showed that recombinant S_1_-, S_2_-, and S_3_-RNases (expressed in *E. coli* and thus non-glycosylated) were all degraded by the 26S proteasome in pollen tube extracts; however, native glycosylated S_3_-RNase purified from styles was not degraded to any significant extent (Hua and Kao, 2006). After purified native S_3_-RNase was deglycosylated, the deglycosylated protein was degraded as efficiently as recombinant S-RNases (Hua and Kao, 2006). Moreover, native S_9_-RNase was ubiquitinated to various extents, with most being mono-ubiquitinated, by an SCF^S7-SLF2^ complex in an *in vitro* ubiquitination assay, but the degradation of the mono-ubiquitinated S_9_-RNase in an *in vitro* protein degradation assay was not efficient (Entani et al., 2014).

It is possible that S-RNase is deglycosylated once taken into the pollen tube. Non-glycosylated S-RNase has been shown to function normally in rejecting self-pollen (Karunanandaa et al., 1994), so deglycosylation should not affect the function of S-RNase. Deglycosylated non-self S-RNase then becomes poly-ubiquitinated and degraded in the cytosol. TTS, a tobacco transmitting tissue glycoprotein, has been shown to be incorporated into the pollen tube wall and deglycosylated (Wu et al., 1995), suggesting that deglycosylation of S-RNase by the pollen tube is possible. It is also possible that poly-ubiquitination and degradation by the 26S proteasome is not the only pathway for detoxification of S-RNase. S-RNase (and deglycosylated S-RNase) may also be mono-ubiquitinated and transported to a compartment for further degradation. Mono-ubiquitination has been shown to play an important role in fast labeling of proteins for bulk degradation by autophagy (Kim et al., 2008). Two plant plasma membrane proteins have been shown to be degraded in the vacuole (Cai et al., 2012). This possible fate of S-RNase can explain two contrasting findings about the destination of S-RNase after its uptake into the pollen tube. Luu et al. (2000) observed that S-RNase of *Solanum chacoense* was mainly localized in the cytosol of both compatible and incompatible pollen tubes, whereas Goldraij et al. (2006) observed that S-RNase of *N*. *alata* was sorted into vacuole-like organelles in both compatible and incompatible pollen tubes. S-RNase may be first taken up into the cytosol of the pollen tubes as observed by Luu et al. (2000), and after the interaction with appropriate SCF^SLF^ complexes (and to a much lesser extent with PiSBP1 and the PiSBP1-containing SLF complex), those non-self S-RNases that are mono-ubiquitinated are sorted to vacuoles or vacuole-like organelles, as observed by Goldraij et al. (2006), for degradation. In the above-mentioned *in vitro* protein degradation assay of Entani et al. (2014), perhaps the compartments might be disrupted and the vacuolar proteases might not be active under the assay conditions, such that no significant degradation was observed for mono-ubiquitinated S_9_-RNase. Self S-RNase may follow similar pathways as non-self S-RNase, except that the bulk of its molecules remain stable due to the inability of any of the SLF proteins to interact with and detoxify it.

## CONCLUSION

In summary, there are several facets of S-RNase-based SI that require further investigation in order to obtain a comprehensive understanding of this complex self/non-self-recognition system. It is clear that S-RNase functions inside the pollen tube to exert its cytotoxicity, but how S-RNase is taken up into the pollen tube is completely unknown. The initial fates of both self and non-self S-RNase after entering the pollen tube also remain unclear. The model presented in Figure 1 depicts several possible pathways for uptake and subsequent fates of self and non-self S-RNases. We believe that the difference in the ultimate fate of self and non-self S-RNase is due in large part to non-self recognition between SLF proteins and S-RNases. A non-self S-RNase interacts with at least one of the SLF proteins and becomes ubiquitinated and suffers degradation, whereas a self S-RNase does not interact with any of the SLF proteins and thus remains stable. The existence of multiple SLF proteins in pollen of any *S*-haplotype and the highly polymorphic nature of the *S*-locus suggest that there are hundreds of SLF proteins (allelic variants of each of the multiple SLF proteins) with a wide range of sequence similarity available for studying interactions with S-RNases. However, so far, the interaction relationships have only been determined between a very small number of SLF proteins and S-RNases. A comprehensive study is necessary to understand the biochemical basis of differential interactions between SLF proteins and S-RNases, and the information obtained will not only further our understanding of S-RNase-based SI, but also lay the foundation for studying the structural basis of the interactions between F-box proteins and their substrates.

### Conflict of Interest Statement

The authors declare that the research was conducted in the absence of any commercial or financial relationships that could be construed as a potential conflict of interest.
